# A catalase promoter variant rs1001179 is associated with visual acuity but not with primary angle closure glaucoma in Saudi patients

**DOI:** 10.1186/1471-2350-14-84

**Published:** 2013-08-20

**Authors:** Khaled K Abu-Amero, Taif Anwar Azad, Ahmed Mousa, Essam A Osman, Tahira Sultan, Saleh A Al-Obeidan

**Affiliations:** 1 Department of Ophthalmology, College of Medicine, King Saud University, Riyadh, Saudi Arabia, PO Box 245, Riyadh 11411, Saudi Arabia; 2 Department of Ophthalmology, College of Medicine, Jacksonville, FL, USA

**Keywords:** Catalase, PACG, Saudi Arabia

## Abstract

**Background:**

To Investigate whether the g.4760C>T polymorphism in the promoter region of the catalase gene (*CAT*) is a risk factor for primary angle closure glaucoma (PACG) in the Saudi population.

**Methods:**

138 unrelated PACG patients and 403 unrelated control subjects from Saudi Arabia were genotyped for a single nucleotide polymorphism (SNP; rs1001179; g.4760C>T) utilizing Taq-Man® assay. The association between different genotypes and various clinical indices important for PACG was also investigated.

**Results:**

The distribution of different genotypes was comparable between both study groups. The genotype “C/C” was predominant among cafses; 94 (68.1%) and controls; 289 (71.7%). Heterozygous genotype “C/T”, was present in 41 (29.7%) of cases and 103 (25.6%) of controls, where the homozygous variant genotype was present in only 3 (2.2%) of cases and 11 (2.7%) of the controls. The distribution of variant allele was similar in both study groups (*p*= 0.568). Interestingly, there was a trend of association between the type of the variant (homozygous variant, heterozygous and wildtype genotype) and one important parameter for PACG, which is visual acuity. The visual acuity increase was; 0.62 (±0.74), 0.88 (±0.88) and 1.27 (±0.95) in patients carrying the “C/C”, “C/T” and “T/T” genotypes respectively, which was statistically significant in both ANOVA and pairwise individual T tests (*p* = 0.022, 0.031 and 0.039) when compared to controls.

**Conclusions:**

This variant is possibly associated with visual acuity in PACG patients and thus had the potential to be used as a parameter for assessing PACG severity.

## Background

Although glaucoma embodies a heterogeneous group of optic neuropathies, all types are defined by progressive and irreversible degeneration of the optic nerve with gradual visual field loss. Within this group, primary angle-closure glaucoma (PACG) is a type of glaucoma characterized by a narrow iridocorneal angle resulting in a blockage of the aqueous outflow structures. It seems that there is an anatomical and physiological predisposition to PACG. Thus, shallow anterior chamber depth is considered an anatomically inheritable risk factor for this illness [1]. Congruently, Eastern Asian ascendance, hyperopia and female gender significantly predispose to this disease [2]. It has been estimated that in Saudi Arabia, 40% of glaucoma patients belong to the PACG type [3]. This percentage is closer to those calculated for Asian than for European populations [4]. Although a hereditary component for PACG exists, causative genes have not yet been identified until recently where a significant association at three new loci: rs11024102 in *PLEKHA7* (P=5.33×10(−12)), rs3753841 in *COL11A1* (P=9.22×10(−10)) and rs1015213 located between *PCMTD1* and ST18 on chromosome 8q (P=3.29×10(−9)) were reported [5]. The three SNPs may explain in part some aspects of the PACG pathogenesis, but not all. Oxidative stress has been implicated to cause increased IOP by triggering TM degeneration and thus contributing to alterations in the aqueous outflow pathway. Indeed, treatment with hydrogen peroxide (H_2_O_2_) impairs TM cell adhesion to the extracellular matrix and causes rearrangement of cytoskeletal structures [6]. In humans, in vivo experiments demonstrated that oxidative DNA damage is significantly more abundant in the TM cells of glaucoma patients. Additionally, both increased IOP and visual field damage were significantly related to the amount of oxidative DNA damage affecting TM cells [6,7]. Oxidative stress is induced through formation of excess reactive oxygen species (ROS). Under normal conditions, ROS is an integral part of signaling pathways; however, when generated in excess lead to formation of oxidative stress on the cellular environment. The ROS that damage cellular macromolecules are scavenged by antioxidant enzymes. Antioxidant enzymes, such as superoxide dismutase (SOD), catalase and glutathione peroxidase have been reported in aqueous humor highlighting their importance to maintain a balanced oxidative status in the eyes. Deficiency or dysfunction of vascular antioxidant enzymes may result in ROS accumulation and health problem ensues. Levels of antioxidant enzymes play an important role in glaucomatous disease. Heritable polymorphisms in the antioxidant enzymes have been evaluated as one of the mechanism affecting the antioxidant's capacity. Catalase is one of the key antioxidant enzymes which reduce hydrogen peroxide to water. Single nucleotide polymorphism (SNP) (g.4760C>T) in the catalase (*CAT*) gene (NG_013339.1) had been shown to lower the catalase activity in the blood [8]. This SNP had been associated with breast cancer risk [9]. Recently we showed that this SNP is associated with three clinical indices important for adult onset primary open angle glaucoma (POAG) [10]. The aim of the present study is to evaluate this SNP as a potential risk factor for PACG in the Saudi population.

## Methods

### Study population

#### Study population

The study adheres to the tenets of the Declaration of Helsinki for research involving humans, and all participants signed an informed consent a priori. The study was approved by the College of Medicine Institutional Research and Ethics Board (approval number # 08–657). Saudi Arab participants with clinically diagnosed PACG and healthy controls were both recruited into the study at King Abdulaziz University Hospital (KAUH) in Riyadh, Saudi Arabia.

We recruited 138 Saudi PACG patients (cases) who satisfied strict clinical criteria for PACG which includes at least three of the following: 1) clinical documentation of angle closure, defined as the presence of appositional or synnechial closure of the anterior chamber angle involving at least 270 degrees by gonioscopy in either eye; 2) intraocular pressure elevated to a level ≥21 mmHg as measured by Goldmann applanation tonometry; 3) evidence of characteristic glaucomatous optic disk damage with excavation of the disc causing a cup-to-disk ratio (c/d) vertically of at least 0.70 in at least one eye; and 4) characteristic peripheral visual field loss including nerve fiber bundle defects (nasal step, arcuate scotoma, paracentral scotoma) or advanced visual field loss (central and/or temporal island of vision) as tested by Humphrey Field Analyzer in those patients with vision better than 20/200 or Goldmann Manual Perimetry in those with worse vision.

Exclusion criteria included: 1) secondary angle closure glaucoma; 2) presence of pseudoexfoliation syndrome even if coexistent with angle closure; 3) another cause of optic nerve injury affecting either eye; 4) significant visual loss in both eyes not associated with glaucoma; 5) inability to visualize the fundus for optic disk assessment; or 6) refusal to participate.

Patients were recruited from the glaucoma clinic at King Abdulaziz University Hospital (KAUH) after signing an informed consent form approved by the institutional review board (proposal number # 08–657).

A second group (n= 403) of healthy Saudi Arabs controls (Controls group) free from glaucoma by examination were recruited. Entry criteria for those subjects were age >40, normal IOP range (≥ 6 and ≤ 21 mmHg), open angles on gonioscopy, and normal optic nerves on examination.

### DNA preparation

DNA from patients and controls was obtained from peripheral blood (7 mL) collected in EDTA tubes from all participating individuals. Extraction was performed using the illustra blood genomicPrep Mini Spin kit (GE Healthcare, Buckinghamshire, UK) and stored at −20°C in aliquots until further use. Quantification of extracted DNA was performed using a NanoDrop ND-2000c spectrophotometer (Thermo Scientific, Wilmington, DE, USA).

### Genotyping and validation for SNP rs1001179 of the CAT gene

Subjects were genotyped to determine the rs1001179 polymorphism (g.4760 C>T) in the promoter region of the *CAT* gene (NG_013339.1) status using the TaqMan® SNP Genotyping Assay (Applied Biosystems Inc., Foster City, CA, USA) on ABI 7500 Real-Time PCR System (Applied Biosystems). The TaqMan assay uses allele-specific fluorogenic probes that, when hybridized to the DNA template are cleaved by the 5’ nuclease activity of the Taq polymerase resulting in fluorescence emission from the reporter dye. Each assay utilizes 2 unlabeled PCR primers and 2 allele-specific probes and each probe is labeled with a different color reporter dye at the 5’ end. In this assay, VIC (for allele 1) and FAM (for allele 2) were used as the reporter dyes. For detection of rs1001179, assay ID: C_11468118_10 was used. The reagents were supplied by Applied Biosystems. Each PCR reaction was performed in a total volume of 25 μL and consisted of 1X TaqMan® Genotyping Master Mix (Applied Biosystems), 1X SNP Genotyping Assay Mix and 20 ng DNA. Each 96-well plate included two no template controls. Real-time PCR was performed on an ABI 7500 using the recommended conditions consisting of incubation at 95°C for 10 min, followed by 40 cycles, denaturation at 92°C for 15 s and annealing/ extension at 60°C for 1 min. The VIC and FAM fluorescence levels of the PCR products were measured at 60°C for 1 min. Analysis of fluorescence using the automated 2-color allele discrimination software on ABI 7500 showed clear discrimination of all genotypes of *CAT* gene on a two-dimensional graph. Validation of real-time PCR genotyping results was performed on 10% of randomly selected samples by sequencing using BigDye terminator v3.1 cycle sequencing kit (Applied Biosystems). Fragments were electrophoresed on the ABI 3130*xl* Genetic Analyzer (Applied Biosystems) according to the manufacturer’s protocol. All of the sequenced fragments were then analyzed using SeqScape software v2.6 (Applied Biosystems).

### Statistical analysis

Genotype and clinical indices data of both cases and controls were collected using a specifically designed data collection sheet including gene profile as well as all potentially associated demographic and clinical indices of patients and controls at presentation. Demographic data included: subject’s age and sex, while clinical data included: medical history for co morbidity of systemic diseases, age at symptoms, duration from onset to presentation, intra-ocular pressure (IOP), cup/disc ratio, Logarithm of the Minimum Angle of Resolution (LogMAR) visual acuity, and the number of current anti-glaucoma medications used. A database was built using Microsoft Access 2007®, where all data were entered, cleaned and managed.

Data analysis was conducted using SPSS version 19.0 by IBM Inc. (Chicago, IL) and StatsDirect version 2.7.2 by StatsDirect Ltd. (Cheshire, UK). The analysis included descriptive statistics where continuous variables were presented as means (±SD) while categorical variables were presented in forms of frequencies and percentages. In terms of inferential analysis, odds ratios, 95% confidence intervals and the corresponding *p* values were used to detect the association between different genotypes and the presence of disease. The student’s T test was used to compare means across groups as regards continuous variables, while Mann Whitney U test was alternatively used whenever indicated. Meanwhile, one way analysis of variance (ANOVA) was used to detect whether there is a difference in clinical indices at presentation among different genotypes (alternatively, Kruskal-Wallis Test was used when indicated). A pairwise t test was also conducted to detect the source of variation between groups when detecting a significant difference using the ANOVA findings. During pairwise comparisons, both heterozygous and homozygous carrying this SNP groups were compared to the group with the wildtype genotype. A threshold *p* value was set to 0.05, where any *p* value < 0.05 was considered as statistically significant.

## Results

A total of 541 subjects in the age interval of 40 years and above were recruited for the current study, where 138 subjects with confirmed diagnosis of closed angle glaucoma were classified as *"*cases*"* and 403 other subjects with confirmed as glaucoma free serving as *"*controls*"*.

The mean (±SD) age for cases was 63.1 (±9.5) years, median 64, range [40–87], while that for controls was 61.1 (±10.8) years, median 60 and range [40–93]. Comparing both age means, there were no statistically significant difference between cases and controls (*p* = 0.054). As regards gender, male cases were 84 (60.9%) compared to 54 (39.1%) females, while in controls, females 282 (70.0%) were higher in number compared to males 121 (30.0); (*p* <0.001). Thirteen (9.4%) patients had a family history of glaucoma, while 39 (28.3%) were diabetic and 35 (25.4%) had hypertension. However, only 17 (12.3%) patients were found to be aware of having glaucoma. Among the 138 glaucoma cases, 131 (94.8%) were bilateral and 7 (5.2%) were unilateral.

With regard to the distribution of the genotypes, the wildtype “C/C” was predominant among both cases; 94 (68.1%) and controls; 289 (71.7%). The heterozygous variant genotype “C/T” was present in 41 (29.7%) of the cases and 103 (25.6%) of the controls, whereas the homozygous variant genotype “T/T” was only present in 3 (2.2%) and 11 (2.7%) in cases and controls respectively. There was no association between having the heterozygous genotype “C/T” and getting closed angle glaucoma; OR: 1.22 [95% CI: 0.773 - 1.197; *p* = 0.372] or homozygous variant “T/T” and having the disease; OR: 0.84 [95% CI: 0.147 – 3.267; *p* = 0.998]. Additionally, the presence of the variant allele “T” was the minority among cases 47 (17.0%) and controls 125 (15.5%) compared to the wild type “C” allele [229 (83.0%)] and [681 (84.5%)] in cases and controls respectively, however, the alleles distribution had no statistically significant association with encountering the disease; [OR: 1.12 (95% CI: 0.756 – 1.6330), *p* = 0.568] (Table 1).

**Table 1:** Distribution of different genotypes among cases and controls

**Genotype**	**Cases**	**Controls**			
	**(n = 138)**	**(n = 403)**	**OR**	**95% CI**	***P *value**
	**No. (%)**	**No. (%)**			
C/C	94 (68.1)	289 (71.7)	-	-	-
C/T	41 (29.7)	103 (25.6)	1.22	[0.773 – 1.197]	0.372
T/T	3 (2.2)	11 (2.7)	0.84	[0.147 – 3.267]	0.998
Allele					
C	229 (83.0)	681 (84.5)	-	-	-
T	47 (17.0)	125 (15.5)	1.12	[0.756 – 1.633]	0.568

All genotypes were in Hardy Weinberg Equilibrium.

Examining the likelihood of an association between genotypes and important glaucoma clinical indices, showed that, the mean (±SD) age at onset increased with change in genotype (homozygous variant is worse than heterozygous and heterozygous is worse than wildtype genotype); 49.0 (±22.7), 57.9 (±12.2) and 62.4 (±8.9) for “C/C”, “C/T” and “T/T” respectively. This increase was however, statistically insignificant in ANOVA (*p* = 0.336). A similar trend was observed in mean (±SD) duration between getting closed angle glaucoma and presentation to the hospital, intraocular pressure (IOP) and logMAR visual acuity. The visual acuity increase was; 0.62 (±0.74), 0.88 (±0.88) and 1.27 (±0.95) in “C/C”, “C/T” and “T/T” groups respectively, which was statistically significant in both ANOVA and pairwise individual T tests (*p* = 0.022, 0.031 and 0.039). More findings on other clinical indices at presentation which were not statistically significant are presented in Table 2.

**Table 2:** Average clinical indices distributed by different genotypes

**Variable**	**C/C**	**C/T**	**T/T**	**All cases**	**Anova *p *value**	**T test *p *value**	
	**Mean (±SD)**	**Mean (±SD)**	**Mean (±SD)**	**Mean (±SD)**	**(All groups)**	**CT/CC**	**TT/CC**
Age at onset	49.0 (22.7)	57.9 (12.2)	62.4 (8.9)	52.9 (18.8)	0.336	0.336	0.351
Duration (Months)	43.1 (45.3)	50.4 (41.4)	53.9 (7.0)	46.7 (42.9)	0.645	0.645	0.690
IOP (mmHg)	18.4 (7.5)	17.6 (7.4)	21.7 (6.8)	18.2 (7.5)	0.397	0.447	0.302
Visual Acuity (LogMAR)	0.62 (0.74)	0.88 (0.88)	1.27 (0.95)	0.71 (0.80)	0.022	0.031	0.039
Vertical Cup/Disc ratio	27.7 (35.9)	28.0 (37.0)	25.6 (49.6)	27.7 (36.2)	0.991	0.957	0.906
Number of medications	1.59 (1.2)	1.41 (1.0)	1.50 (0.8)	1.50 (1.2)	0.647	0.356	0.854

*LogMAR* Logarithm of the Minimum Angle of Resolution.

*IOP* Intraocular pressure.

## Discussion

Our interest in studying *CAT* rs1001179 polymorphism in the settings of glaucoma stems is due to the following reasons: **First**, catalase is an important enzyme for maintaining the oxidative status of the body and plays a very important role in protecting the eukaryotic cells from going into premature apoptosis. Catalase is a common enzyme found in nearly all living organisms exposed to oxygen. It catalyzes the decomposition of hydrogen peroxide to water and oxygen. The *CAT* gene encodes catalase, a key antioxidant enzyme in the body's defense against oxidative stress. Catalase is a heme enzyme that is present in the peroxisome of nearly all aerobic cells. Catalase converts the ROS hydrogen peroxide to water and oxygen and thereby mitigates the toxic effects of hydrogen peroxide. Oxidative stress is hypothesized to play a role in the development of many chronic or late-onset diseases such as diabetes, asthma, Alzheimer's disease, systemic lupus erythematosus, rheumatoid arthritis, and cancers. Polymorphisms in this gene have been associated with decreases in catalase activity but, to date, acatalasemia is the only disease known to be caused by this gene [11,12]. **Second**, the profound effect of the g.4760C>T sequence change on the catalase level in the blood [8]. **Third**, the increasing evidence of the link between glaucoma and mitochondrial abnormalities [13] and the pivotal role mitochondria plays in survival and maintenance of cellular homeostasis of the RGCs [14]. **Fourth**, the link between oxidative stress and glaucoma via the apoptotic events at the RGCs [13]. And **lastly**, our recent study had shown that this SNP is associated with three clinical indices important for adult onset POAG [10]. Considering all these factors we investigated the possible association of a SNP (rs1001179) in the promoter of CAT gene with POAG patients of Saudi origin. To the best of our knowledge, this SNP role in glaucoma pathogenesis was not previously investigated in the literature apart from our previous study aforementioned [10].

The results presented in Table 1, indicated that genotype frequencies between cases and controls were almost similar and there was no statistically significant difference between the two groups. When we investigated the variant “T” allele frequency in the cases and the controls, we found no difference (*p* = 0.568). This indicates that neither the variant genotype nor the variant allele is a risk factor for PACG in this population. It’s possible that the link between mitochondrial abnormalities and/or oxidative stress and glaucoma are not linked through the catalase pathway and that other oxidative stress pathway(s) may be involved. In any case, the exact mechanism of how oxidative stress contributes to glaucoma pathogenesis remains speculative. Additionally, glaucomatous optic neuropathy implies loss of RGCs, including their axons, and a major tissue remodeling, especially in the optic nerve head. Mechanisms leading to glaucomatous optic neuropathy are not yet clearly understood. We previously investigated mitochondrial abnormalities in a group of patients with PACG and found absence of mitochondrial abnormalities in those patients [15]. We suggested at the time that optic nerve injury in PACG has been attributed primarily to elevated IOP caused by anatomic changes in the anterior and posterior globe [16], in contrast with the molecular and biochemical abnormalities suspected in POAG [17].

Interestingly, when we investigated the potential association between the presence of a certain genotype and different clinical indices, important for PACG, we found an interesting trend corresponding to various clinical indices and severity of the variant (i.e. Homozygous variant, followed by heterozygous, and the least severe is the wildtype). The mean (±SD) age at onset increased with increase in variation severity (homozygous variant is worst than heterozygous and heterozygous is worst than wildtype), but statistically insignificant. On the other hand, the visual acuity increase was; 0.62 (±0.74), 0.88 (±0.88) and 1.27 (±0.95) in “C/C”, “C/T” and “T/T” groups respectively, which was statistically significant in both ANOVA and individual T tests (*p* = 0. 022, 0.031 and 0.039 respectively). Other clinical indices listed in Table 2 were not associated with the genotypes investigated.

There are few limitations to this study: i) the study was conducted on the Saudi population only and thus need to be carried out in other populations to establish the link between this variant and various clinical indices; 2) at this point, modifiers for this catalase SNP leading to glaucoma are not known and thus we will not know for sure if this SNP alone can influence the disease severity; 3) We cannot exclude other SNPs at the same locus, which may be associated with POAG and 4) Considering the fact that the frequency of the homozygous (T/T) variants was low (3/138; 2.2% in cases and 11/403; 2.7% in controls) it is also plausible that more individuals are needed for such case–control genetic association study.

## Conclusion

In summary, we showed a possible association of the catalase polymorphism (g.4760 C>T) with the visual acuity, which is an important clinical indices for PACG. Thus, this SNP can be potentially used as a marker for PACG severity.

## Abbreviations

CAT: Catalase; c/d: Cup-to-disk ratio; IOP: Intraocular pressure; KAUH: King Abdulaziz University Hospital; LogMAR: Logarithm of the minimum angle of resolution; PACG: Primary angle closure glaucoma; POAG: Primary open angle glaucoma; PCR: Polymerase chair reaction; ROS: Reactive oxygen species; RGCs: Retinal ganglion Cells; SNP: Single nucleotide polymorphism; SOD: Superoxide dismutase; TM: Trabecular meshwork.

## Competing interests

The authors declare that they have no competing interests.

## Authors’ contributions

KKA supervised the overall study, writing the manuscripts and analysis of the data; TAZ and TS carried out the technical work in the laboratory; AM statistical analysis of the data and writing the manuscripts; EAO and SAO patient's requirements and reviewing the files for clinical information. All authors read and approved the final manuscript.

## Pre-publication history

The pre-publication history for this paper can be accessed here:

http://www.biomedcentral.com/1471-2350/14/84/prepub
